# Internet Addiction and Quality of Life in College Students: A Multiple Mediation Analysis

**Published:** 2019-11

**Authors:** Tingting GAO, Yu-Tao XIANG, Zeying QIN, Yueyang HU, Songli MEI

**Affiliations:** 1. Department of Social Medicine and Health Management, School of Public Health, Jilin University, Changchun, China; 2. Unit of Psychiatry, Institute of Translational Medicine, Faculty of Health Sciences, University of Macau, Macao SAR, China

## Dear Editor-in-Chief

Excessive or problematic use of the Internet has been a worldwide phenomenon and is increasingly more common among young people. Internet addiction (IA) is an impulse control problem and characterized by an inability to inhibit Internet use that has an adverse impact on major life domains (1). People who spend more time communicating their inner thoughts to other people on the Internet are more likely to have a lower level of life quality (2). Internet search activity was closely associated with the suicide rate (3). The potential impact of the Internet on suicidal behavior was prominent as a public health concern (4). Individuals with suicidal behaviors may display worse quality of life (5). Excessive Internet usage tends to become poor sleepers. Short sleepers had poorer quality of life, which has been getting attention (6).

The aim of this study was to investigate the mediating roles of suicidal behaviors and duration of sleep in the relationship between Internet addition and quality of life. A cross-sectional survey was conducted in April 2016, using a self-reported questionnaire. It employed convenience sampling method to produce a sample of college students. Approximately 781 questionnaires were delivered to participants. A total of 701 valid questionnaires (498 females) were completed in the survey. The age of participants ranged from 16 to 25, with an average of 20.50 years (SD=1.42).

The study was approved by the research ethics board of Jilin University. All the invited participants were voluntary and the consent form was obtained.

**Fig. 1:**
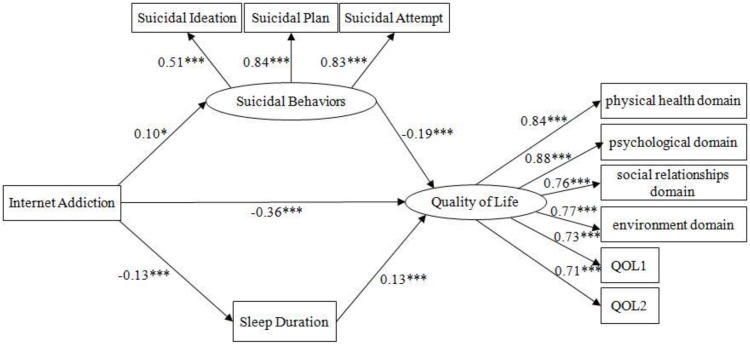
The path coefficients of multiple mediation models

Data analyses were carried out by Amos 17.0 and SPSS 18.0. Structural equation modeling was adopted to test the multiple mediation models. The results for this modified model showed a very good fit, χ^2^/df=2.99, GFI=0.98, CFI=0.98, NFI=0.98, TLI=0.97, RESEA=0.05. The path coefficients in the Fig. 1 were standardized regression coefficients and significant at the level of *P*<0.05. To confirm the mediation effects, we applied the bootstrapping analysis (number of bootstrap samples=5000) by the corresponding SPSS macro to measure the significance of each indirect effect (Table 1). The results showed there was a parallel mediating effect of suicidal behaviors and duration of sleep on the association of Internet addiction and quality of life. Suicidal ideation occurs when an individual has a sense of thwarted belongingness and perceived burdensomeness. However, The Internet can meet the need to establish a sense of belonging. Not everyone who has suicidal ideation will commit suicide. Some of them might try to acquire capability for suicide. The accessibility of suicide-related information on the Internet seems to have an impact on the incidence of suicide behaviors. Besides, higher levels of suicidal behaviors would be significantly associated with worse quality of life. College students with Internet addiction would spend more time on Internet use, resulting in less sleep duration. Then the quality of life will be lower. Excessive Internet use may have not only direct adverse quality of life, but also have indirect negative effects through sleep deprivation.

**Table 1:** Bootstrap analysis of the significance test of multiple mediation effect

***Paths***	***Effect***	***SE***	***95%CI***
Indirect effect 1			
Internet addiction ➛ Suicidal behaviors ➛ Quality of life	−0.04	0.01	(−0.07, −0.02)
Indirect effect 2			
Internet addiction ➛ Duration of sleep ➛ Quality of life	−0.02	0.01	(−0.03, −0.01)
Total indirect effect	−0.06	0.01	(−0.09, −0.03)
Direct effect			
Internet addiction ➛ Quality of life	−0.36	0.04	(−0.44, −0.29)

The conclusion of our study further enriches the mechanism of the effect of Internet addiction on quality of life. It also provides the basis for improving the quality of life of college students.
